# Sport Promotion through Sport Mega-Events. An Analysis for Types of Olympic Sports in London 2012

**DOI:** 10.3390/ijerph17176193

**Published:** 2020-08-26

**Authors:** Themistocles Kokolakakis, Fernando Lera-Lopez

**Affiliations:** 1 Sport Industry Research Centre, Sheffield Hallam University, Sheffield S1 1WB, UK; 2 Institute for Advanced Research in Business and Economics, Department of Economics, Public University of Navarra, 31006 Navarra, Spain; lera@unavarra.es

**Keywords:** sport participation, London Olympic Games, Olympic legacy, sport legacy, trickle-down effect, sport events, active life, age, gender, sport promotion

## Abstract

A substantial amount of attention has been devoted towards the potential sport legacy of the Olympic Games. In spite of the increasing academic interest in this topic, there is a knowledge gap as far as sport legacy is concerned by types of different sports. The authors bridge this gap by analysing the evolution of 43 different Olympic/Paralympic sport modalities in the two-year period after the London 2012 Olympics. By using data from the Active People Survey with a sample of 165,000 people annually, and considering some demographic variables and the effect of the economic environment, the paper aims to test the existence of a sport legacy. We have applied time series analysis and ARIMA models for controlling for economic influence and seasonal adjustment and for making comparisons among participation rates. The results show, for the total of the sports analysed, that there were 336,000 individuals who increased their frequency of participation, while there was no significant increase in the number of new participants in these sports. When we develop the analysis for types of sports, London 2012 is positively associated not only with the frequency of participation in some types of sport but also with an increase in the number of new sport participants. Gender and age differences are also detected. The results show the differences of sport legacy by type of sports. Moreover, this research has elucidated an important unrecognised aspect of the effect of the Olympic Games and perhaps major events: that they can become a major policy tool for reversing sporting inequalities.

## 1. Introduction

A substantial amount of attention and debate has been devoted during the last few years towards the potential sport legacy effects of the Olympic Games. This interest is closely associated with the need to increase sport and physical activity (PA) levels and reduce the sedentary behaviour of individuals. Recent evidence has shown that people do not undertake enough PA and that PA levels are falling in many countries [1]. Globally, the World Health Organization (WHO) argues that inactivity rates have been rising from 23% in 2010 to 27.5% in 2016 [2] with at least $67.5 billion of economic burden annually [3]. In Europe, the proportion of those who say they never exercise or play sport has increased between 2009 and 2017 to 46% [4] leading to one million premature deaths and corresponding to 10.4% of all deaths in Europe [2].

Nevertheless, despite the social interest in this field of study, there is a knowledge gap when considering the sport Olympic legacy due to the mixed results obtained by previous empirical evidence. From a theoretical perspective, sport Olympic legacy considers four types of direct and indirect effects [5,6]. Firstly, hosting the Olympic Games may have a positive effect on the population in the host country in terms of increasing the desire of individuals to be involved in an enjoyable event (festival effect) and/or inspiring individuals to participate for the first time in sports or increase the frequency of participation among existing participants (demonstration effect) [7,8,9]. Secondly, people may be inspired to participate by elite athletes as a result of their personalities and popularity (“role models”), although on the other hand, they may deter participation because the standard of performance of elite athletes may be seen as impossible to emulate [10]. These conflicting effects may explain some of the mixed results obtained [11]. Thirdly, elite sporting and national success in international competitions have a positive inspiration effect on the population, contributing to some people becoming sport participants [9]. Indirect effects include other potential effects that could influence sport participation [5] through instruments such as improvement in sport infrastructure and transportation [12] and promotion and coverage of the progress for a sport event [13].

From an empirical approach, systematic reviews made by Weed et al. (2012, 2015), Mahtani et al. (2013), and McCartney et al. (2010) have found no reliable evidence to confirm that any previous Olympic Games had succeeded in encouraging and increasing sport participation rates for the hosting nations [8,9,14,15]. These reviews show how the sport legacy is more likely to materialise in terms of increasing the participation frequency or in activity switching (demonstration effect) rather than increasing the number of participants [13].

Nevertheless, some studies have suggested some short-term positive effects (i.e., Truño (1995) for Barcelona 1992 [16]; Veal (2003) and Veal et al. (2012) for Sydney 2000 [12,17]; Georgiadis and Theodorikakos (2016) and Pappous (2011) for Athens 2004 [18,19]; Potwarka and Leatherdale (2017) for Vancouver 2010 [20]; Chen and Henry (2016) and Kokolakakis et al., (2019) for London 2012 [21,22]) and long-term positive effects (Aizawa et al., (2018) for Tokyo 1968 [5]). Others have demonstrated no relationship (i.e., Bauman, Bellew, and Craig (2015) for Sydney 2010 [23]; Feng and Hong (2013) for Beijing 2008 [24]) whereas some others have found differences among different types of sports (Veal (2003) and Veal et al. (2012) for Sydney 2000 [12,17]).

These differences highlight some research shortcomings such as the lack of longitudinal studies, limited differentiation of population groups and the exclusive use of qualitative instruments in some studies. The lack of longitudinal data and/or appropriate population-level data, together with differences in sample sizes and changes in survey design, make it difficult to compare the results obtained [12]. This is clear when comparing empirical evidence about London 2012. For example, studies with small sample sizes have found an increase of motivation to take part in sports and an increase in amount of exercise, particularly among individuals that were already engaged in sport [21,25,26]. In contrast, other studies considering the evolution of sport participation rates with data from official and national surveys, such as the Active People Survey (APS) have obtained mixed results. For example, Henry (2017) describes a decrease in sport participation rates from October 2012 to March 2015 with significant differences among age and socio-economic groups [27]; while Grix et al. (2017) show a decline on sport participation rates post-2012 to almost the same levels evident in 2005 [28]. Downward, Dawson and Mills (2013) found that during the Games, there was a drop (10%) in sport participation, particularly in Olympic sports, arguing that watching the Games was a substitute good for practising sport [29]. Recently, Kokolakakis et al. (2019) have showed an increase in the percentage of the population that participated at least three times a week in the year immediately after the Games [22]. This result corroborates previous arguments about the legacy being less relevant to new participants than to existing participants who as a result increase their participation [8,30].

Some authors have emphasised the differences among sports and consequently the potential legacies could be associated with the type of sport under analysis [31]. For example, Pappous and Hayday (2015) estimated the impact of the 2012 London Olympic Games on participation rates in judo and fencing. They concluded that an increase in participation occurred in these sports, comparing 2007 and 2013 [32]. Brown, Essex, Assaker, and Smith (2017) analysed the most popular sport in the UK, swimming, with a survey of 316 individuals during and immediately after the Games, making the distinction between individuals attending the event live or watching the event on television. They concluded that people attending the live event were more likely to participate in swimming in the future than people watching the event on TV [33]. Grix et al. (2017) analysed 10 Olympic disciplines from 2005/6 to 2014/15 using the information provided by the Active People Survey (APS). They showed significant differences among the 10 sports [28]. While there were significant increases in participation rates in five sports—athletics, cycling, boxing, table tennis, and netball (with differences in increasing rates)—there was a clear decrease in swimming, less significant declines in badminton, volleyball and hockey, and no significant change in gymnastics.

The purpose of this study is to offer new insights into the sport legacy of the Olympic Games by analysing 43 different sport Olympic and Paralympic modalities of the sport legacy of London 2012 classified into three groups: (1) combat Olympic sports, (2) team Olympic sports, and (3) water-based Olympic sports, in the two-year period immediately following the Games. Specifically, we examine if there has been a step change in these sports in terms of participation among adults in England using data from the Active People Survey (APS). To the best of our knowledge, until now no research has considered a large set of different sports in the Olympic Games. In addition, compared with previous research, we have broadened the number of sports under study, we have considered a two-year period after the Games and we have applied ARIMA models to make comparisons among participation rates. If hosting Olympic Games has a sport legacy, then mega sport events could be a tool for boosting sport participates rates, thereby leading to positive effects on health and subjective well-being outcomes at the population level. An analysis for type of sports might help policymakers when hosting sports events in the future to develop specific legacy plans.

## 2. Materials and Methods

The research was conducted by analysing the dataset provided by the eight waves of the APS between 2005 (first year of the APS) and 2014. We considered quarterly sport participation rates for three dimensions of frequency for the 43 sports under analysis. Applying ARIMA models, we forecasted the expected participation rates using the pre-Olympic participation trend adjusted for seasonality and changes in GDP. By comparing the real participation rates with the expected/forecasted participation emanating from the pre-Olympic trend, we estimated the sport legacy in each sport. This section illustrates both the data and methodology used to evaluate any changes in sport participation following the London Olympic Games 2012.

### 2.1. Data

The APS was the largest survey of sport and active recreation in Europe; its cycle ran from mid-October continuously for 12 months. Around 165,000 English adults (age 16 and over) were interviewed annually by telephone across the country. The sample was randomly stratified, and the results were representative of the total adult population in the country. The large size of the survey makes it ideal to explore the associations between sport participation and the demographic profile of the population in England [34]. There are no ethical issues to report; every respondent provided informed consent, and all data were anonymised.

For the purpose of this research, sport participation data were collected from the fourth quarter of 2005 to the third quarter of 2014 (36 data points). The logic was to monitor the development of sport participation before and around the Games. Quarters were selected (rather than months) in order to facilitate the use of the seasonally adjusted GDP figures from the National Accounts, which are also published on a quarterly basis. This, in turn, was accompanied by a seasonal adjustment of participation rates in order to smooth the data and to remove the seasonal effect. In doing so, comparisons could be established between changes in GDP and participation rates without the distortions of seasonal effects.

We have constructed an overall variable for general participation, but at the same time we have constructed variables for three types of Olympic sport participation and we have also considered the analysis of four sports with a high sport participation in the UK: swimming, athletics, equestrianism and cycling. In particular, we considered 43 Olympic/Paralympic sports, following the definitions provided by Sport England [35], as being the most relevant: Archery, Athletics, Badminton, Basketball, Boccia, Boxing, Canoeing, Cycling, Equestrian, Fencing, Goalball, Handball, Hockey, Judo, Modern Pentathlon, Rowing, Sailing, Shooting (monthly only), Swimming, Table Tennis, Taekwondo, Tennis, Triathlon, Volleyball, Weightlifting, Wheelchair Basketball, Wheelchair Rugby, and Wrestling. These sports have been classified into three groups:

- Combat Olympic sports: Boxing, Fencing, Judo, Taekwondo, Wrestling.
- Team Olympic sport: Basketball, Goalball, Handball, Hockey, Volleyball, Wheelchair Basketball, Wheelchair Rugby. The Olympic/Paralympic and Team Olympic sport groups do not include football for two reasons: (i) the Olympics is not as big an event in the male football calendar as other major international events; and (ii) the national allegiance of UK football fans tends to be with their home nation rather than a Great British and Northern Irish team.
- Water-based Olympic sports: Canoeing, Rowing, Sailing.

Based on the APS, the previous variables were constructed considering participation in sport of adults aged 16 and over (as early APSs had age 16 as their starting point) in three different dimensions of frequency for the 43 Olympic/Paralympic sports under analysis:

3 × 30: The proportion of adults participating in at least 30 min of sport, at moderate intensity, on at least 12 days out of the last 28 days (equivalent to 3 or more days a week).
1 × 30: The proportion of adults participating in at least 30 min of sport, at least moderate intensity, on at least 4 days out of the last 28 days (once a week).
1 × m: The proportion of adults participating at least once a month for at least 30 min of sport, at least moderate intensity.

We assume that new participants might start with different levels of frequency and that actual participants might increase their level of frequency; it is often assumed that a way to detect new participants is to observe rises in the 1xm participation rates [22].

Furthermore, since participation in sport is affected by some demographic factors [36,37], we examined the participation rates overall and in demographic groups including gender, age intervals, and disability. Table 1 illustrates these variables and all the sport participation categories following the London Games. Around 7% of the English population have participated at least three times a week in Olympic/Paralympic sports, increasing to 27.4% of the population for a monthly participation. The most-practised Olympic sport in the period of analysis is swimming, followed by cycling and athletics. Furthermore, in terms of demographic differences, men participate more than women and increases in age are associated with declines in participation rates, as suggested by previous empirical evidence.

**Figure 1:**
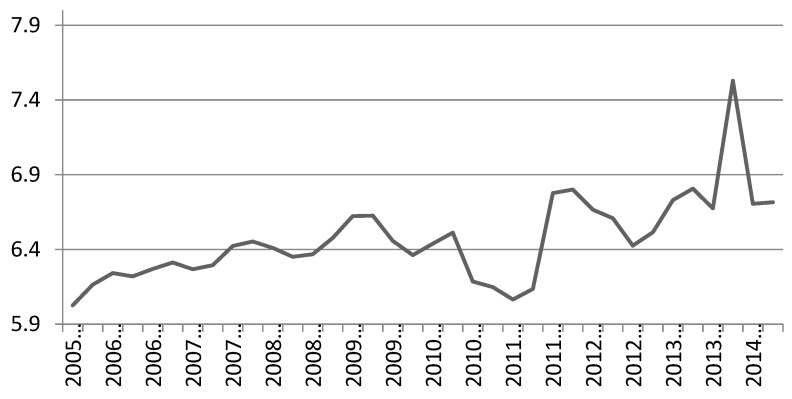
Olympic sport rates, 3 × 30 definition, seasonally adjusted participation.

**Figure 2:**
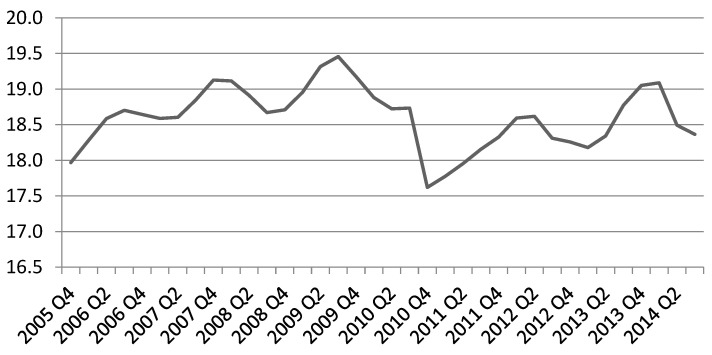
Olympic sport rates, 1 × 30 definition, seasonally adjusted participation.

To elucidate better the legacy effect as it appears by the seasonally adjusted participation rates, we present two graphs for the participation rates of the Olympic sports under the definitions of 3 × 30 and 1 × 30 (see Figure 1 and Figure 2 below). It is evident that there is a visible legacy under the 3 × 30 definition, even without adjusting for economic activity (as it is the case in those two graphs). This immediately indicates the validity of the research that Olympic and major sport events affect the frequency of participation as illustrated in [9,22]. Notice also the dips in participation rates associated with the recession of 2009–2010 which must be taken into account in the remaining calculations.

### 2.2. Methodology

Most research work deals with participation on an annual basis. When we move away from the annual observations to quarterly or monthly observations, the existing seasonality has to be removed. In the case of sport rates, the dominant seasonal pattern sees participation picking up in the third quarter (summer) and dropping strongly in the fourth (winter). However, the seasonality pattern is of little interest to the objective of this research, obscuring the real effect as we move from one quarter to another.

In general, four steps were followed:

1. Derivation of actual quarterly participation variables from APS for the period 2005–2014;
2. Seasonally adjusting the participation rates for the categories described in Table 1 over time;
3. Derivation of expected participation rates, using seasonally adjusted GDP and the pre-Olympic sport participation trend, for the period 2012–2014;
4. The difference between the actual seasonally adjusted participation rates (from the APS in step 2) and the expected participation rates (using the aforementioned step 3) provides a measure of the Olympic association with sport participation (2012–2014).

From the above steps, the methods of seasonal adjustment and deriving expected participation rates need more detailed explanation. Firstly, seasonal adjustment was conducted using a combination of Excel spreadsheets and specialist data analysis software (SPSS (IBM, Armonk, NY, USA) and X-12-ARIMA (U.S. Census Bureau, USA)). The X-12-ARIMA seasonal adjustment package (developed by the United States Bureau of Census (2007) and freely available from their website) was chosen for de-seasoning the participation data. This software is used by the British Office for National Statistics [38] as the standard software for official statistics. The seasonal adjustment was done using an optimum ARIMA model that was chosen automatically from the software package, on the basis of work by Gómez and Maravall (1998) [39]. The chosen moving average process (though the internal process of the software) at the heart of the seasonal adjustment was ARIMA (010) (011). This approach ensured consistency in adjustment between participation and GDP. For ease of comprehension, note that the first part of the ARIMA model (the first bracket) is the non-seasonal part, while the second is the seasonal part (in this case over quarterly data). Here, the non-seasonal part is defined by a random walk, while the seasonal part by a single seasonal difference and a single moving average term. The first number in each bracket indicates the autoregressive process (AR, lagged dependent variable), the second the degree of difference (Integration) used, and the third the moving average process (MA, the regression error expressed as a linear combination of error terms). Through the ARIMA model we generate as much as possible a stationary series which eases the calculation process and it has been previously applied to the analysis of the sport participation Olympic legacy [22].

Secondly, the participation rates trend (for 2012–2014) was estimated by using seasonally adjusted GDP at constant (2011) prices and a time trend. Based on previous literature, if there is a sport legacy, this would be apparent in the full year of the Olympic Games and thereafter. This time-frame (2012–2014) for the effect was tested in this research through the examined dataset for all the detailed variables. We developed a pre-Olympic regression to calculate a trend for sport participation without the effect of London 2012, whilst simultaneously abstracting from the effects of changes in GDP and sport seasonality. Including GDP was important because of the association between income and sport participation, evident in all aforementioned research and the 2009–2010 economic recession which had the strength to potentially change the structure of sport participation. Previous studies have tried to control for the influence of this significant variable in the analysis of the sport participation legacy [5,6,22].

The chosen model is adopted by Sport England from Gratton and Kokolakakis (2012) [40], and it has participation regressed on a constant, a time trend and the percentage change of GDP three quarters before, across all indicators. For example, in the case of the 3 × 30 swimming definition this model becomes:

P_t_ = 1.62 − 0.01xt + 0.002x∆G_t-3_ (0.03) (0.002) (0.01) R2 = 58%, standard errors in brackets,(1)

where P and ∆G stand for percentage of swimming participation and percentage change in GDP (between successive quarters) respectively. The regressions for each sports participation category are available upon request.

The time period for the regression is from 2005 Q4 to 2011 Q4. This time period creates a model that can help us trace the trend of sport participation in the subsequent period. The advantage of this approach is that the participation data set is seasonally adjusted in exactly the same way as national GDP and that in the formation of the expected participation rate (2012–2014), GDP is taken explicitly into account following the greatest recession of recent times.

To illustrate the accuracy of the forecast, we employed the mean absolute percentage error statistic. This is a useful way to communicate forecasting data because the result is expressed in percentage terms, which are more meaningful in the context of our research. The expression giving the mean absolute percentage error (MAPE) is:

$$\mathrm{MAPE}=\;\overset{T}{\underset{t=1}{\sum }}\frac{\left|Y_{t}-\hat{Y_{t}}\right|}{Y_{t}T}\;\times 100$$

This measure of accuracy can only be applied in the pre-Olympic period, as during the Olympic year and after we are relying on diversions from the forecast to evaluate the Olympic legacy. By using the existing model to forecast the participation rates for the recessionary period 2009–2010 (eight quarters), MAPE gives diversion values of 1.4% and 1.2% for the 3 × 30 and 1 × 30 definitions correspondingly. Given that this is the period that participation rates dipped out of trend because of the recession, (see Figure 1 and Figure 2) the results reassure for the forecasting strength of the model.

## 3. Results

Table 2 and Table 3 present the results for each variable under examination:

- Total sport legacy effect: the sum of participation gains (or losses) in all quarters of 2012–2014, comparing, in percentage terms, the actual and expected participation curves. We calculate this effect for three frequencies: 3 × 30, 1 × 30 and 1 × m.
- Percentage point sport legacy effect (2012–2014): the total sport legacy effect (above) in all quarters of 2012–2014, compared to the participation rates of the period 2009–2011 for the three frequencies. As every sport and type of sport could have a historically different sport participation rate, with this variable we compare the sport legacy effect with previous historical participation rates for each of the sports during the period 2012–2014. Furthermore, we estimate this percentage for the individual years 2012–2014, in order to check the sustainability of the effect during the years following the Olympics.
- The number of extra participants (associated with the Games legacy) in the average quarter of the period 2012–2014 can be derived, coupled with derivations of the sport effect per year.

We have divided the analysis into two different parts. Firstly, we analyse the existence of the sport legacy effect for different groups of Olympic sports, and secondly, we analyse four popular Olympic sports: swimming, athletics, equestrianism and cycling.

### 3.1. Differences by Sport Groups

This section describes the sport legacy effect in the Olympic sports and in three types: Team sports, Combat sports, and Water-based sports. In general, the sport legacy effect is for the 3 × 30 category and to a lesser extent for the 1 × 30 category, while there is no effect for 1 × m. As it is shown in Table 2, in the period 2012–2014, compared to the pre-Olympic level, there was a 5.38% increase in participation for the intensive 3 × 30 definition (with 150,000 individuals increasing their frequency of participation) and a 2.30% for the 1 × 30 variable (186,000 people). However, there was no effect in the 1 × m category, meaning that overall the sport legacy effect associated with the Olympic Games is stronger when the frequency of participation increases. 

In Table 2 above, the first column describes the sum of gains in participation over the years 2012–2014 as differences between the actual and the expected participation rates for the period 2012–2014. The greater the value, the more profound the effect that can be associated with the Olympics. However, such a statistic is of little use for the comparisons we want to make. If we start from a participation rate of 30% it is much easier to have a 2% gain to 32% than if we start from a small base such as 4%. In the latter case, a 2% points gain to 6% would represent a 50% increase in participation. For this reason, we transform the absolute gains of the first column, expressing them in the second column as percentages of increase in existing participation rates. This statistic is a better way to evaluate the association of the Olympic Games with each category of sports and it has been also estimated for every year under examination. The same approach has been developed to analyse sport legacy in some sports and by sociodemographic variables (see Table 3 and Table 4 below).

Note that when we examine the sport legacy effect by types of Olympic sports, the results vary significantly. For example, in the case of Combat sports, we have the first major reversal of the basic participation pattern—in this case, the percentage point effect of 2012–2014 becomes greatest under the 1 × m definition (8.23%) and there is a sizeable impact of 21,000 additional participants in Combat sports associated with the Games.

The sport legacy effect for the low frequency of participation (1 × m variable) is also evident in Team and Water-based sport participation, increasing by 20.66% and 16.98% respectively. Clearly, the London Games changed significantly the number of people interested in these sports, which combined with a relatively small base of participants, gave a very strong percentage point effect and a significant increase in the numbers of participants of 105,000 for Team sports and 64,000 for Water sports. Furthermore, in both cases the percentage change in 3 × 30 participation is very high, at 26.68% and 79.38% correspondingly. In fact, the Water-based sports 3 × 30 category gave the strongest percentage point sport legacy effect.

In terms of comparing the sport legacy effect during the period under analysis, in general, results show an increase in the strength of the effect in 2014 compared to 2013 and 2012 when hosting the Games. This is true in each case, except in Water sports 1 × m and 3 × 30 variables, where the values are lower than in 2012.

Finally, in terms of increases of sport participants, the greatest number is in the Olympic 1 × 30 case (186,000). From the subcategories, the greatest effect occurred in the Team sports 1 × m (105,000).

### 3.2. Differences in Some Individual Sports

Table 3 examines four popular individual sports in the UK: Swimming, Athletics, Cycling, and Equestrianism. Swimming is the only sport with its 1 × 30 percentage effect being greater than the 3 × 30 one. The 1 × 30 participation rate increased by 3.90% compared to the pre-Olympic period, which means 122,000 participants in this sport at least once a week. There was no impact in the 1 × m definition, implying that no significant number of participants started swimming during the examined period.

Both in Equestrianism and in Athletics, the main sport legacy effects were detected in the intensive 3 × 30 definition; in the case of Equestrianism, this was the only effect. Their participation rates, over the 2012–2014 period, increased by 5.75% and 7.50% correspondingly, with 44,450 and 13,494 individuals increasing their frequency participation to at least 3 × 30. In the case of Athletics, most of the effect occurred in 2014 and 2012, while in Equestrianism the percentage point effect in 2013 was the greatest, exceeding 13%. Finally, in Cycling the percentage point sport legacy effect on participation was 4.92% and 4.36% in the 3 × 30 and 1 × 30 definitions correspondingly, with 26,591 and 83,344 new participants in these intensities. There was no effect in the 1 × m category, as it has happened with the other sports.

Table 4 illustrates the gender, age intervals, and disability effects on sport legacy. In terms of gender, the sport legacy effect is higher among women for all the three sport participation frequencies under examination. In fact, in men, a sport legacy only exists among regular practitioners. In terms of age intervals, the sport legacy effect is higher among people under 34 years and above 64 years. The legacy is in general more important for regular practitioners than for less regular participants (1 × m) in Olympic sports; furthermore, there is an increase of regular practitioners in Paralympic sports.

## 4. Discussion

The analysis of the sport participation legacy has become a recurrent one in academic discourse since the International Olympic Committee (IOC) introduced it as its 14th mission [41]. In the case of the London 2012 Games, for example, the sports legacy was a core component of the wider legacy of the Olympic Games in the UK [42]. In fact, London 2012 was the first Olympic Games to explicitly define and attempt to deliver this type of legacy [43]. In a context of stagnation of PA and sports participation rates in many countries [4], this has justified an increase in the number of studies analysing the sport participation legacy of the Olympic Games.

The systematic reviews presented in the literature section did not find reliable evidence for increases in sport participation, giving mixed results. In general, they showed a positive short-term increase in the participation frequency rates rather than a rising in the numbers of participants. Nevertheless, analyses for types of sports have been generally neglected with some exceptions [12,28].

In our research we have considered 43 different Olympic/Paralympic sport modalities in the two-year period after the London 2012 Olympic Games, applying time series analysis for controlling for economic influence and to make comparisons among participation rates. The results show, for the total of the sports analysed, that there is an increase in terms of frequency of participation, with a total of 336,000 individuals who have increased their frequency, while there was not a significant increase in the number of low-frequency participants of these sports. This is supported by previous studies that argued that the Olympic Games are more likely to boost sport participation frequency and re-engagement of lapsed participants in regular participation, than to increase the number of new sport participants with low frequency [7,8,9,22,30,31,44].

Nevertheless, when we develop the analysis for types of sports, we obtain that London 2012 is positively associated not only with regular participation in some types of sport but also with an increase in the number of less regular participants, in particular in combat Olympic sports, with an increase of 8.23% and 21,000 participants at least once a month, confirming evidence shown by Pappous and Hayday (2015) for judo and fencing [32]. In other types of more popular sports such as team and water sports, the number of participants at least once a month is 105,000 and 64,000 respectively. Moreover, in both cases, the sport legacy effect for the intensive participation is 26.68% and 79.38%, respectively.

When we consider some of the most popular individual Olympic sports in the UK such as Swimming, Athletics, Cycling, and Equestrianism, we conclude there is no sport legacy effect for non-regular participants and that in general the effect is focused on the most intensive participation (3 × 30), with values higher than for the overall participation rates in the total Olympic sports. Swimming is the only sport with its 1 × 30 percentage effect being greater than the 3 × 30 one. The 1 × 30 participation rate increased by 3.90% compared to the pre-Olympic period, implying an increase of 122,000 participants practising this sport at least once a week. It is possible that the predominance of 1 × 30 swimming is due to its place within the Health and Fitness industry, with swimming being one of several activities one can undertake. This result partially contradicts previous evidence on a large decrease in weekly participation in swimming after the Games [28]; in our case the weekly effect (3 × 30) showing an increase in frequency is less pronounced than the weekly effect showing an increase in participation (1 × 30).

Another important result that we have obtained for practically all the types of sports under study is the fact that the sport legacy effect is in general higher the second year after the Games than immediately after the Games. This result, indirectly, emphasizes the importance and relevance of the sport legacy planning in order to obtain an increase in sport participation rates after hosting the Olympic Games [28,45]. In fact, until the London Games, no sustained participation programs were implemented to generate a sport participation legacy [8,46]. Comparative analysis has confirmed that sport participation legacies could be achieved if hosting governments are able to engage the society and coordinate efforts between different government levels and a wide range of stakeholders in long-term strategies [47]. In the case of the London Games, programmes and policies were developed to boost sport participation rates, which could be associated with the positive sport effects obtained in this study. In the case of Rio Olympic Games, the legacy proposed was based on the idea of social development through sport, but according to Reis, de Sousa-Mast, and Gurgel (2014) and Reis et al. (2017), the lack of legacy planning after the bid was won and the allegations of corruption made against the Minister of Sports in 2011 were barriers to an effective implementation of policies to produce the sport legacy [47,48].

Finally, analysis by demographic variables of the London sport legacy for the Olympic and Paralympic sports shows some differences. In terms of age intervals, we confirm previous empirical evidence [20,22,49] about a higher increase in participation among young people around the Olympic Games, in particular among regular participants. Compared to other age intervals, people over 64 years also show an important increase in participation across the three definitions used in this research. This means that, among older people, London 2012 has attracted not only regular practitioners but also new and less regular participants for the Olympic/Paralympics sports under consideration. Similar conclusions could be applied to females compared to males.

The results of this research can lead to important conclusions for sport policy. According to these results, the Olympic Games might have the potential to reverse existing inequalities in sport use. This can be taken seriously into account in policy matters around sport in the actual context of the Covid 19 epidemic. According to Sport England (2020), the coronavirus is disproportionately affecting certain groups of people such as people with disabilities and minority ethnic communities [50]. In our research, females and aged people near and over 64 years old show percentage gains in participation much higher than any other group under consideration. These results can also be extended to the case of people with disabilities: their response to the Olympic Games is clearly higher than the youngest age category (16–34) under consideration. Hence this research has elucidated an important unrecognised aspect of the effect of the Olympic Games and perhaps major events: that they can become a major policy tool for reversing sporting inequalities.

There are also lessons for investment policy, associated with any influx of new participants. The amount of generated consumer spending is greater when new participants are introduced into a sport compared to existing participants that increase their participation. A new participant usually buys a full set of sportswear and equipment generating higher sport-related Gross Value Added and income for the Government through indirect taxation. Consequently, the identified sports where there is a considerable rise in participation on a weekly or monthly basis can better justify any possible demand for increased investment. Furthermore, as every sport requires different equipment and clothes, an analysis by sport could have different implications for the sport industry. Additionally, the fact that people above 64 years are encouraged by the Olympic Games to practise more regularly could have significant economic implications as they enjoy more leisure time.

It is important to recognise the limitations of our study, some of which have given rise to areas of future research. As noted by Coalter (2007) [51] or more recently by Aizawa et al. (2018) [5], it is difficult to draw definitive conclusions regarding the impact of the Olympic Games on sport participation because a simple and unique cause and effect cannot be established. Other external variables might influence this relationship. In this context, for example, it is not possible to isolate the association of the Olympic Games in some sports with some events of these sports in England which have shown a positive association with an increase in participation levels under the period of analysis [44]. Furthermore, this study has been restricted to the two years following the Olympic Games. Although a short-term impact has been established, further analysis should be developed to check the longer-term impact of hosting the Olympic Games. Note also that unless used for panel analysis, the APS is a cross-sectional dataset rather than a longitudinal one, making it impossible to trace changes in individual sports behaviours [27]. However, it should be expected that the further we move away from the Games, the more difficult it will become to associate effects with the event. Furthermore, it is not possible to develop a counterfactual analysis, considering trends in sport participation in the period 2012–2014 in other countries. Reasons include the fact that the 2008 recession had a different impact on the economic structure of each country; furthermore, many countries do not measure quarterly sport participation rates for different types of sports.

## 5. Conclusions

This paper offers new insights into the sport legacy of the Olympic Games by analysing 43 different sport Olympic and Paralympic modalities from London 2012, classified into three groups: (1) Combat Olympic sports, (2) Team Olympic sports, and (3) Water-based Olympic sports. Compared to previous research, we have broadened the number of sports under study, we have considered a two-year period after the Games and we have applied econometric techniques to make comparisons among participation rates. The results show, for the total of the sports analysed, that there were 336,000 individuals who increased their frequency of participation, while there was not a significant increase in the number of less regular participants in these sports. When we develop the analysis for types of sports, London 2012 is positively associated not only with the frequency of participation in some types of sport but also with an increase in the number of infrequent sport participants: in the case of combat Olympic sports, an increase of 8.23%, equivalent to 21,000 new participants, was observed. In swimming, 122,000 new participants are engaged in sport at least once a week. Another important result that we have obtained for practically all the types of sports under study is the fact that the sport legacy effect is in general higher the second year after the Games than immediately after the Games. Finally, analysis by demographic variables of the London sport legacy shows a higher increase in participation among females, disabled people, young people and people over 64 years. To sum up, this paper shows that, when focusing the analysis on individual sports, London 2012 has attracted not only regular practitioners but also non-regular participants for the Olympic/Paralympics sports under analysis, promoting active life among English people and highlighting an important unrecognised aspect of the Olympic Games: that they can become a major policy tool for reversing sporting inequalities.

## Figures and Tables

**Table 1:** Sport Participation Rates (2012 Quarter 4–2014 Quarter 3) by sport categories and demographic variables (%, thousands: th).

Frequency of Participation:	3 × 30 (%)	3 × 30 (th.)	1 × 30 (%)	1 × 30 (th.)	1 × m (%)	1 × m (th.)
Overall	25.2	11,007	44.3	19,349	53.0	23,149
Olympic/Paralympic Sport Group (No Football)	6.6	2883	18.4	8037	27.4	11,968
Combat Olympic Sports	0.2	87	0. 5	218	0.6	262
Team Olympic Sports (No Football)	0.1	44	0.7	306	1.2	524
Water based Olympic Sports	0.1	44	0.4	175	1.1	480
Swimming	1.4	611	6.7	2926	11.4	4979
Athletics	1.9	830	4.6	2009	6.5	2839
Cycling	1.3	568	4.6	2009	8.2	3582
Equestrian	0.4	175	0.7	306	0.9	393
Females	21.7	4852	39.9	8922	48.4	10,823
Males	29.0	6155	49.0	10,427	57.9	12,327
Age: 16–34	34.8	4728	58.0	7880	68.8	9348
Age: 35–54	27. 5	4047	48.3	7109	58.1	8551
Age: 55–64	19.7	1197	36.3	2205	43.8	2660
Age: 65+	10.7	1035	22.3	2156	27.0	2590
Disability (restricting)	12.3	770	24.2	1515	30.9	1934

**Table 2:** Sport legacy effect by Olympic sport groups.

	Total Sport Effect (2012–2014)	Percentage Point Sport Effect (2012–2014),	Extra Participants in Average Quarter (2012–2014)	Percentage Point Sport Effect (2012)	Percentage Point Sport Effect (2013)	Percentage Point Sport Effect (2014, q1–3)
Olympic 3 × 30	4.13	5.87	150,000	3.60	4.30	8.89
Olympic 1 × 30	4.68	2.30	186,000	1.05	2.41	3.90
Olympic 1 × m	none	none	none	none	none	none
Combat 3 × 30	0.11	7.01	4000	none	11.66	11.96
Combat 1 × 30	0.14	2.78	6000	0.12	2.77	5.84
Combat 1 × m	0.52	8.23	21,000	3.04	7.36	14.91
Team 3 × 30	0.39	26.68	15,000	14.19	20.70	50.70
Team 1 × 30	1.71	22.71	68,000	7.18	14.75	53.88
Team 1 × m	2.64	20.66	105,000	9.18	15.68	43.49
Water 3 × 30	0.72	79.38	29,000	83.66	55.23	61.18
Water 1 × 30	0.90	21.56	36,000	17.60	17.05	30.81
Water 1 × m	1.61	16.98	64,000	17.41	18.32	13.25

**Table 3:** Sport legacy effect in some Olympic sports.

	Percentage Point Sport Effect (2012–2014)	Total Sport Effect (2012–2014)	Extra Participants in Average Quarter (2012–2014)	Percentage Point Sport Effect (2012)	Percentage Point Sport Effect (2013)	Percentage Point Sport Effect (2014, q1–3)
Swim 3 × 30	3.10	0.49	19,000	3.94	4.99	none
Swim 1 × 30	3.90	3.07	122,000	3.59	6.89	1.02
Swim 1 × m	none	none	none	none	none	none
Athletics 3 × 30	5.75	1.12	44,450	5.58	1.77	9.96
Athletics 1 × 30	none	none	none	none	none	none
Athletics 1 × m	none	none	none	none	none	1.38
Cycle 3 × 30	4.92	0.67	26,590	1.98	6.89	5.69
Cycle 1 × 30	4.36	2.10	83,344	1.99	5.43	5.96
Cycle 1 × m	none	none	none	none	none	none
Equestrian 3 × 30	7.50	0.34	13,494	2.72	13.32	6.71
Equestrian 1 × 30	none	none	none	none	none	none
Equestrian 1 × m	none	none	none	none	none	none

**Table 4:** Sport legacy effect in Olympic sports by demographics (All Olympic and Paralympic sports).

	Percentage Point Sport Effect (2012–2014)	Total Sport Effect (2012–2014)	Extra Participants in Average Quarter (2012–2014)	Percentage Point Sport Effect (2012)	Percentage Point Sport Effect (2013)	Percentage Point Sport Effect (2014, q1–3)
Females 3 × 30	12.49	25.36	516,000	7.66	18.20	10.88
Females 1 × 30	5.32	21.63	440,000	2.58	8.99	4.27
Females 1 × m	3.35	16.66	339,000	0.52	8.47	0.52
Males 3 × 30	9.97	27.14	526,000	5.19	15.39	8.83
Males 1 × 30	none	none	none	none	1.93	none
Males 1 × m	none	none	none	none	0.93	none
Age 16–34 3 × 30	11.74	37.79	467,000	6.29	19.19	9.20
Age 16–34 1 × 30	2.38	14.36	177,000	0.80	5.97	none
Age 16–34 1 × m	1.10	7.92	98,000	none	5.11	none
Age 35–54 3 × 30	8.37	21.99	294,000	5.60	13.43	5.08
Age 35–54 1 × 30	2.24	11.31	151,000	0.80	5.54	none
Age 35–54 1 × m	0.38	2.34	31,000	none	4.38	none
Age 55–64 3 × 30	14.62	26.51	146,000	10.48	18.92	13.11
Age55–64 1 × 30	0.80	3.03	17,000	none	3.98	none
Age 55–64 1 × m	none	none	none	none	0.73	none
Age 65 + 3 × 30	21.32	20.29	172,000	13.52	23.84	25.05
Age 65 + 1 × 30	10.15	21.75	184,000	4.85	15.30	10.38
Age 65 + 1 × m	8.29	21.55	182,000	3.13	15.54	5.92
Disability 3 × 30	11.86	12.60	72,000	13.78	15.23	3.41
Disability 1 × 30	3.59	8.08	46,000	4.15	8.47	none
Disability 1 × m	none	none	none	0.42	6.09	none
